# Traumatic Closed Proximal Muscle Rupture of the Biceps Brachii in Military Paratrooper

**DOI:** 10.1155/2019/3472729

**Published:** 2019-08-29

**Authors:** Georgios Kalinterakis, Emmanouil Antonogiannakis, Ioannis Rampakakis, Evangelos Tsialogiannis, Athanasios Syllaios, Miltiadis Ziogas

**Affiliations:** ^1^First Department of Orthopedics, 401 General Military Hospital Of Athens, Athens 11525, Greece; ^2^First Department of Surgery, General Hospital of Athens “Laiko”, National and Kapodistrian University of Athens, Athens 11527, Greece

## Abstract

Traumatic closed proximal muscle rupture of the biceps brachii has been infrequently cited in the medical bibliography. Early reports of this injury derived from US military during parachute jumps, and it may compromise >4% of injuries at altitude. The mechanism is a direct blow to the upper extremity by static lines. We report a case of traumatic closed proximal rupture of the biceps brachii in a healthy 25 years of age military paratrooper. He was managed with primary surgical repair, and after three years of follow-up, the patient has excellent functional results.

## 1. Introduction

Traumatic closed proximal muscle rupture of the biceps brachii has been infrequently cited in the medical bibliography. This injury is chiefly associated with military static line parachute jumps where the paratrooper orients the static line incorrectly around his arm at the onset of jumping causing a straight blunt force on the biceps brachii [1]. Moreover, United States of America is the only country which has reported this specific static line injury [2].

We report a case of traumatic closed proximal rupture of the biceps brachii in a healthy 25 years of age military paratrooper.

## 2. Case Report

A 25-year-old right-handed paratrooper was presented to orthopedic emergency department because he felt a sudden sharp pain in his right upper extremity after attempting a parachute jump.

**Figure 1:**
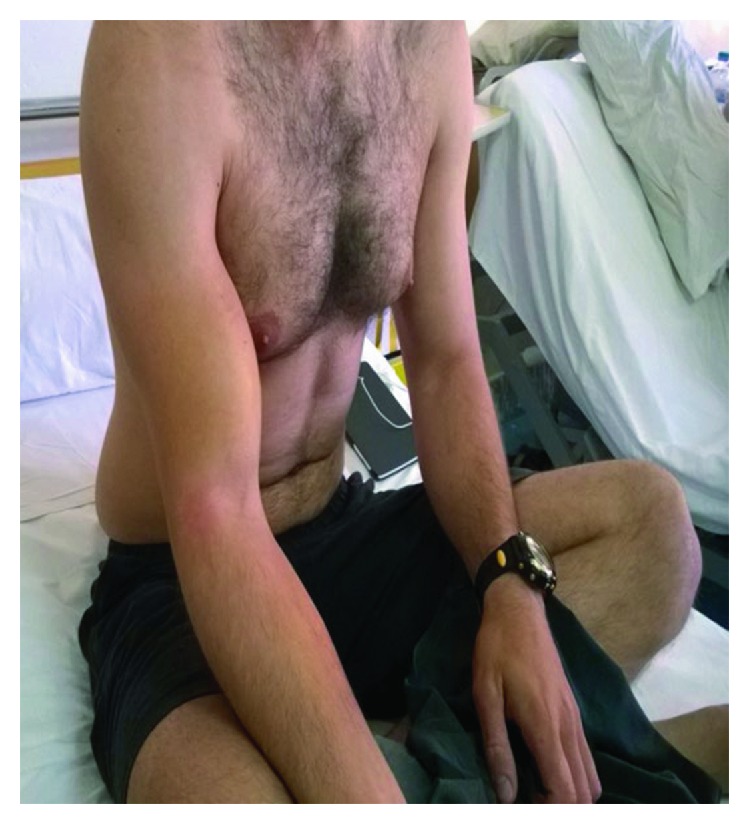
Acute biceps rupture in a military parachutist. Popeye deformity is noted.

On physical examination, there was pain around the right shoulder and upper arm. Open trauma was not observed. Furthermore, extensive ecchymosis and edema were presented due to subcutaneous hemorrhage with spreading in the forearm. The muscle defect was palpable, and Popeye's deformity was noticed as shown in Figure 1. Additionally, supination of the forearm and elbow flexion strength was evaluated in comparison with the uninjured limb. Both decrease in motor strength and weakness in elbow flexion were noted. Manual strength testing revealed 4/5 strength of elbow flexion and supination. Neurovascular examination of the afflicted upper extremity was negative.

**Figure 2:**
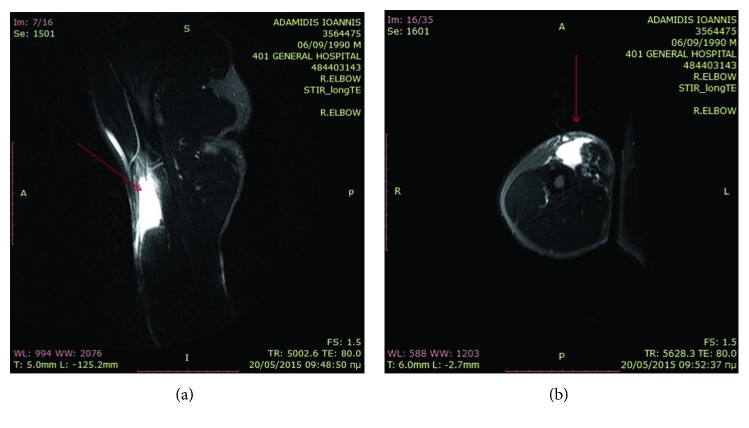
(a, b) MRI images showing an intrasubstance tear of the biceps brachii (arrows) with a large hematoma filling the defect.

Simple radiographs did not reveal bony pathology. Magnetic resonance imaging (MRI) of the right upper limb disclosed the proximal rupture of the biceps brachii at the musculotendinous junction (Figures 2(a) and 2(b)).

**Figure 3:**
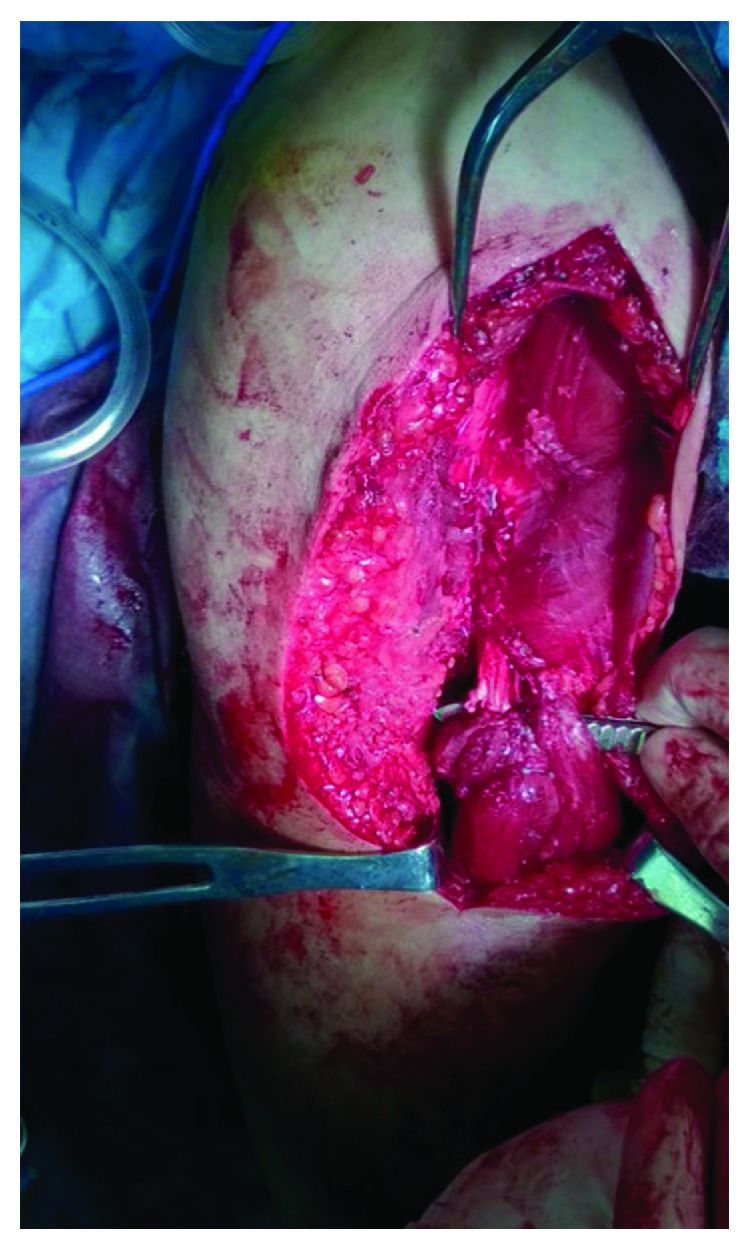
Intraoperative photograph showing the intramuscular biceps tear (yellow arrows).

**Figure 4:**
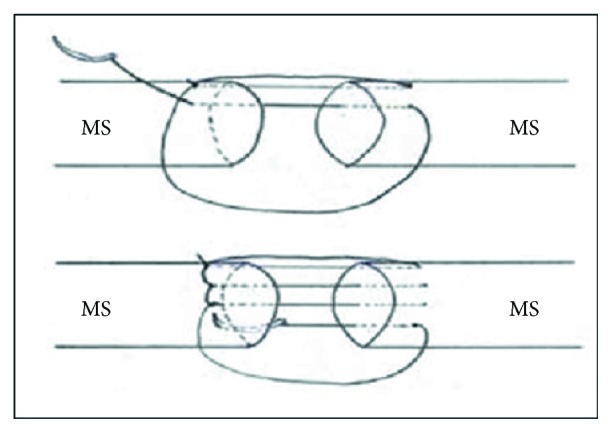
Locking intramuscular sutures secure both the proximal and distal muscle segments (MS).

On the grounds that the patient was both active and young, operative treatment was decided in order the deformity be diminished and the strength be restored. The latter plays a prominent role in case of highly demanded occupation. The surgical intervention was undergone 10 days after the injury. Under general anesthesia, the patient was placed in beach chair positioning and an anterior approach was preferred. A lazy “S” 10-12 cm incision was made with a proximal medial edge on the deltopectoral groove and ending with a distal midline edge on the lateral aspect of the humerus, about halfway down its shaft. When the subcutaneous fascia was opened, a great hematoma of roughly 300 cc was moved out. As a result, better visualization and mobilization of the muscle was achieved. After that, damaged tissue was carefully removed and muscle belly ends were thoroughly inspected. On observation, the long head of the biceps brachii was almost ruptured beneath the myotendinous junction and the short head was completely transected within the intramuscular substance (Figure 3). The musculocutaneous nerve was identified in the interval between the biceps and the brachialis muscles and carefully protected. Then, the gap was bridged with locking intramuscular nonabsorbable sutures (number 2, ETHIBOND EXCEL braided polyester on a tapered needle) (Figure 4). During that process, the elbow flexed to 90° and the forearm positioned in neutral. With the upper extremity in that position, the defect was diminished and the surgical repair was aptly facilitated. Lastly, the trauma was closed in layers.

**Figure 5:**
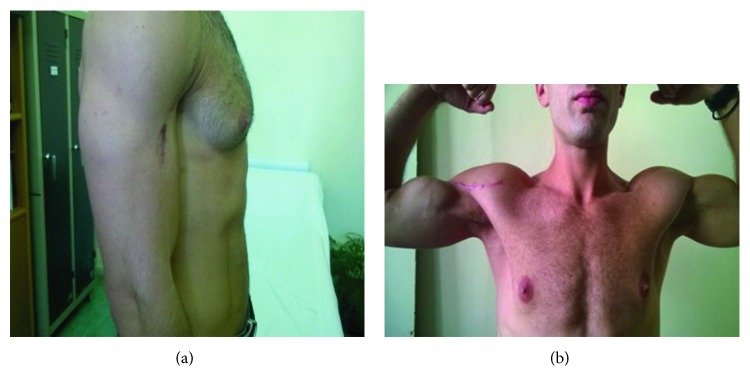
Postoperative images three years after the surgery.

Postoperatively, a posterior splint with the elbow at 90° was used for a period of 4 weeks. When the splint was removed, active ROM exercises based on gravity were started. The DASH score when the splint was removed was 64.2. At 8 weeks, physiotherapy was begun emphasizing on strengthening exercises. Altogether, 20 sessions were done and reevaluation was followed after one month. The DASH score was 16.7. The patient was able to fully return to former activities in six months' time. The DASH score was 0.0. The next follow-up was scheduled one year after the injury. After that, it was recommended that he should be examined once a year. The last follow-up that took place 3 years after the injury revealed great functional results with a full return of strength as well as satisfactory cosmetic results (Figures 5(a) and 5(b)). The patient did not mention any difficulties in his labor and everyday life activities.

## 3. Discussion

Closed proximal ruptures of the biceps brachii muscle are rare incidents, and they usually involve the long head of the biceps. Early reports of this injury derived from US military during parachute jumps, and it may compromise >4% of injuries at altitude [3].

The most common mechanism is a direct blow to the upper extremity by static lines. It is claimed that the static line becomes misrouted under the arm at the time of exit from the aircraft, and this in turn ends up with a blunt trauma to the biceps belly [4]. In our case, the jump platform was a C130 military aircraft. The parachute used was a MC1-1C maneuverable parachute assembly designed by the United States Army in 1988 for military static line airborne operations. This type of injury occurs in the phase of the exit. As the paratroopers approach the door, they hold the static line in their right hand in the case of exiting the right door (as in our case) or in their left hand in the case of exiting the left door. When they reach the door, the paratroopers hand off the static line to the jumpmaster and forcefully adduct their arms holding the reserve parachute. During the free fall, the body of the paratrooper is in a horizontal position for some seconds because of the speed and direction of the aircraft. At the same time, the static line unfolds and finally becomes taut, pulling the deployment bag, in which the parachute is packed, and allowing the canopy to inflate. In the unfortunate event of misrouting of the static line, a localized force may be exerted on the anterior surface of the arm and cause injuries to the upper extremity. This is exactly what happened in our case, according to the description of the patient. The onset of the pain was in the first seconds of the exit and continued to exist for the rest of the fall [3, 5].

The largest series biceps muscle belly tears have come from Womack Army Medical Center in Fort Bragg, NC. Heckman and Levine reported 48 patients in 1978 [1] and Kragh and Basamania reported 12 patients in 2002 [6]. Outside of military parachutists, biceps muscle belly tears are only reported as individual cases [7–9]. Imaging may include ultrasonography and magnetic resonance imaging (MRI). The latter seems to be useful for the detection and characterization of this injury while ultrasound has been reported as an adjunct to MRI in both diagnosis and determination of injury severity [4]. In this case, MRI of the right upper limb revealed the injury confirming its essential role in diagnosis.

Treatment can be either operative or nonoperative. Early reports suggested operative exploration and primary repair [10]. Currently, there is a scarcity of randomized, controlled studies to support standardized operative or nonoperative treatment while few treatment comparison studies have been performed (Table 1). Heckman and Levine compared operative repair vs. early percutaneous hematoma evacuation and immobilization, but no significant difference in clinical outcomes was found [1]. On the contrary, Kragh and Basamania demonstrated better results with operative repair when it comes to strength, appearance, and patient satisfaction [6]. Although no comprehensive data exists to support operative vs. nonoperative treatment, the present case was treated surgically with excellent postoperative results. With regard to postoperative immobilization and rehabilitation, recommendations have varied including elbow immobilization at ≥90° of flexion from 3 days to 3 weeks postoperatively, followed by the use of a dynamic splint for early motion [6, 7]. Although in this case a posterior splint with the elbow at 90° was used for a period of 4 weeks, there were no problems in terms of the rehabilitation program with good functional outcomes in the long term. Finally, the duration of follow-up in most of the studies was short while in some of them the outcome was completely omitted [4].

In conclusion, closed proximal tears of the biceps brachii comprise a rare injury. Military static line parachute jumps seem to be the predominant mechanism. As a result, orthopaedic surgeons must be aware of this clinical feature especially if they work in military hospitals. This study corroborates the superiority of surgical repair in a long term basis and acts as a contributory factor in medical literature in terms of guiding treatment.

## Figures and Tables

**Table 1:** The table demonstrates the published studies regarding the traumatic intrasubstance rupture of the biceps brachii, the type of treatment, and their results.

Study	Treatment	Outcome
Conwell [11]	Open, debridement, no repair, splinting	Good outcome reported
Gilcrest [10]	Surgical repair with primary reattachment using catgut suture and fascial flap	Unreported
Tobin et al. [12]	Unreported	Unreported
Heckman & Levine [1]	Nonoperative group: hematoma aspiration, splinting for 6 weeks in acute flexion. Operative group: open, debridement, primary repair, splinted in acute flexation for 4 weeks, then 90° flexation for 2 weeks, followed by gentle ROM	53% return of elbow flexion in the nonoperative group vs. 76.5% in the operative group. One wound infection was reported. No complications in the nonoperative group
Mellen [13]	Open, exploration, hematoma evacuation, excision of intraluminal thrombus, brachial artery grafting and repair, no biceps repair performed	Skin breakdown and superficial wound infection reported. Visible and palpable defect persisted
DiChristina & Lustig [7]	Open, primary repair, 3 weeks in splint	Full ROM, 5/5 strength at 4 months
Bricknell [14]	Closed, 4 weeks in sling	Visible and palpable defect persisted, no functional deficit compared to contralateral side
Balkissoon et al. [15]	Nonoperative	Unreported
Craig & Lee [3]	Unreported	Unreported
Kragh & Basamania [6]	Nonoperative: sling & NSAIDs. Operative: open, debridement, primary repair, 3-5 day immobility followed by dynamic splinting with extension limited to 30° with early active ROM	All patients returned to full ROM. No complications, job changes, or poor functional outcomes reported. Patients disliked the Popeye deformity. Nonoperative patients regained 65% of contralateral supination/flexion compared with 89% of those repaired operatively
Shah & Pruzansky [9]	Open, debridement, primary repair, postoperative splinting for 4 weeks, followed by hinged bracing for 6 weeks	Full ROM, 5/5 strength at 5 months
Carmichael et al. [8]	Open, debridement, no repair	Full ROM, 5/5 strength at 8 months
Chen & Chew [16]	Nonoperative, cast immobilization for 6 weeks in hyperflexed supinated position	Satisfactory cosmetic and functional results were reported

NSAIDs: nonsteroidal anti-inflammatory drugs, ROM: range of motion.
